# Revisiting Dynamical Theory To Elucidate Friedel’s Law Breaking in Low-Energy Electron Diffraction as Strong Evidence of Unidirectional Growth of Monolayer 2H MoS_2_

**DOI:** 10.1021/acs.nanolett.5c05097

**Published:** 2026-01-09

**Authors:** Dohoon Kim, Joohee Oh, Chaehyeon Ahn, Joonbyeong Jeon, Hyeeree Joo, Hyunseob Lim

**Affiliations:** † Department of Chemistry, 65419 Gwangju Institute of Science and Technology (GIST), 123 Cheomdangwagi-ro, Buk-gu, Gwangju 61005, Republic of Korea; ‡ Center for Quantum Conversion Research, Institute for Basic Science (IBS), Gwangju 61005, Republic of Korea; § GIST InnoCORE AI-Nano Convergence Institute for Early Detection of Neurodegenerative Diseases, Gwangju Institute of Science and Technology (GIST), 123 Cheomdangwagi-ro, Buk-gu, Gwangju 61005, Republic of Korea; ∥ Department of Semiconductor Engineering, Gwangju Institute of Science and Technology (GIST), 123 Cheomdangwagi-ro, Buk-gu, Gwangju 61005, Republic of Korea

**Keywords:** unidirectional growth, low-energy electron diffraction, dynamical theory, Friedel’s law, non-centrosymmetric 2D materials

## Abstract

Unidirectional growth of monolayer molybdenum disulfide (MoS_2_) holds immense promise for next-generation 2D electronics, yet robust and facile characterization techniques to verify its single-crystal characteristics at the wafer scale remain elusive. Although 3-fold symmetric low-energy electron diffraction (LEED) patterns have been presented as evidence of such growth, their fundamental origin and precise link to MoS_2_ orientation have not been clearly understood. Here, we revisit dynamical theory to elucidate Friedel’s law breaking in LEED, providing a comprehensive understanding of energy-dependent LEED intensities that uniquely confirm unidirectional growth of the monolayer 2H MoS_2_. By systematically acquiring LEED intensity–voltage (*I*–*V*) curves, we reveal that the distinct intensity asymmetries observed in symmetry-related diffraction spots directly reflect the non-centrosymmetric characteristic of the MoS_2_ monolayer, amplified by dynamical scattering. This approach allows an unambiguous determination of the monolayer orientation, addressing a critical gap in the qualitative interpretation of LEED.

## Graphical abstract

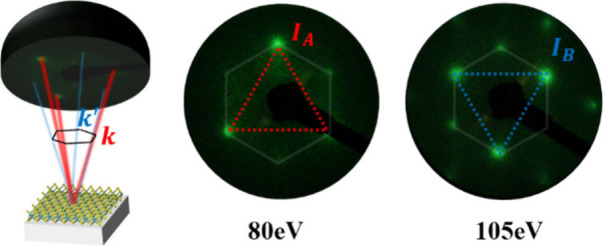

Two-dimensional transition metal dichalcogenides (TMDs) have emerged as promising materials for next-generation electronic, optoelectronic, and photonic devices, owing to their tunable band gaps, strong light–matter interactions, and feasibility of wafer-scale integration.^1–9^ To fully exploit these properties, it is essential to achieve and verify unidirectional growth, where all single-crystal domains share the same in-plane orientation. Because most monolayer TMDs are non-centrosymmetric, 0° and 180° twin domains can form concurrently during growth and can produce distinct crystal orientations that are not superimposable,^9–11^ giving rise to differences in valley-selective optical transitions, anisotropic charge transport, and heterostructure interface matching.^12–17^ Coexistence of opposite orientations inevitably generates grain boundaries, which degrade both structural quality and device performance.^18–24^ Significant progress in chemical vapor deposition (CVD) methods has enabled the large-area synthesis of high-quality monolayer TMDs, including wafer-scale unidirectional single-crystal domains.^10,11,23–29^ These developments reduce grain misalignment and defect densities and open the pathways for systematic investigation for anisotropic properties of TMDs. However, verifying the perfect unidirectionality remains challenging. When triangular single-crystal flakes are spatially isolated, one can often determine their orientation, whether “up” or “down”, by direct imaging.^11,24,30^ Once the domains coalesce into a continuous monolayer, this becomes far more difficult, as conventional characterization techniques typically cannot differentiate between inverted orientations in a non-destructive manner.^5,31–33^ As a result, films containing mixed orientations are sometimes misinterpreted as single crystals.

While a variety of standard analytical tools are available for probing basic structural features, many fail to resolve inverted orientations in fully merged films. Low energy electron diffraction (LEED) offers a powerful, non-destructive approach for examining surface symmetry and long-range order in 2D crystals.^33–37^ In TMD systems, the appearance of a 3-fold LEED pattern has often been presented as evidence of unidirectional growth.^24,25,38–40^ This pattern reflects a breaking of Friedel’s law,^41–43^ which states that the diffraction intensity for a reflection (*h*, *k*, *l*) is equal to that for (−*h*, −*k*, −*l*), leading to asymmetries in intensities between (*h*, *k*) and (−*h*, −*k*) spots. While such symmetry breaking has been extensively exploited in transmission electron microscopy (TEM) techniques, including convergent-beam electron diffraction (CBED) and dark-field TEM, and has also been observed in prior LEED studies of asymmetric structures, its systematic application for quantifying orientation distributions in coalesced 2D TMD monolayers has remained largely unexplored.^44–47^ However, the presence of a 3-fold LEED pattern alone does not prove perfect unidirectionality. It can also arise when domains of opposite orientations are present in unequal proportions, producing an apparent but not absolute orientation bias. Without careful quantification of LEED spot intensities and their variation with incident electron beam energy, it is difficult to unambiguously determine the true orientation distribution. Herein, we address this issue using monolayer molybdenum disulfide (MoS_2_) as a representative TMD system. MoS_2_ is ideal for orientation studies because of its direct band gap (∼1.8–1.9 eV)^1–4^ and the availability of scalable CVD methods that yield large-area, high-quality single crystals.^25,48–50^ Through systematic LEED measurements, we analyze the physical origin of diffraction intensity asymmetries, investigate their dependence on electron energy, and quantitatively determine orientation distributions. Our findings not only clarify the structural origin of the 3-fold symmetry in MoS_2_ but also establish a generalizable diffraction-based framework for verifying unidirectional growth in other TMDs, including WS_2_, MoSe_2_, and WSe_2_, as well as in van der Waals heterostructures. Furthermore, this quantitative framework for a single-crystalline monolayer serves as a fundamental study for distinguishing layer thickness and complex stacking sequences (e.g., ABA vs ABC), which requires rigorous energy-dependent analysis beyond single static imaging to resolve subtle symmetry variations. This approach provides a rigorous pathway to confirm wafer-scale single crystallinity and to guide the controlled synthesis of high-performance 2D semiconductors for device applications.

Recent significant advancements in TMD growth by the CVD method,^25,27,50–53^ including our previous works,^54^ have enabled unidirectional growth of various TMDs, such as MoS_2_ and WS_2_. This unidirectional growth is generally attributed to unequal adsorption energies of orientation at 0° [120*n*° (*n* = 0, 1, and 2) denoted as A] and orientation at 180° [60 + 120*n*° (*n* = 0, 1, and 2) denoted as B] on single-crystal stepped vicinal substrates, which favor alignment in one predominant orientation. We have recently summarized and compared various characterization techniques for analyzing unidirectional growth of TMDs in our review,^31^ providing a framework for selecting appropriate methods depending on the growth stage and sample form. The most convincing evidence for this phenomenon has traditionally been the direct observation of triangular domains aligned in the same direction before they merge into a continuous film.^24,30,53^ However, for practical applications, it is essential to demonstrate unidirectionality even after these domains coalesce into a full film, necessitating a reliable method for verifying the orientation in a fully merged sample. Polarized Raman spectroscopy,^55^ photoluminescence (PL),^4,56^ and second harmonic generation (SHG) measurements are well-known to exhibit orientation-dependent signals in TMD materials.^5,52,57–59^ However, because both incident and detected light remain unaffected by an inverted symmetry, it is generally not possible to distinguish A from B; i.e., these techniques can easily differentiate an orientation angle such as 0° from 30°, while the orientations of 0° (A) and 60°, 180°, or 300° (B) appear effectively identical. These spectroscopic methods can confirm the coexistence of the A and B domains only indirectly by identifying the grain boundaries between them. While X-ray diffraction cannot distinguish inverted orientations of non-centrosymmetric crystals, electron diffraction can distinguish between them owing to the different types of diffraction sources, radiation (X-ray) and charged particle (electron).^33,41,60^ To study LEED of single-crystal MoS_2_ (*sc*-MoS_2_) and epitaxially grown MoS_2_ with nearly equal populations of A and B orientations (*ep*-MoS_2_), two different types of monolayer 2H MoS_2_ films were synthesized using an inorganic molecular CVD (*im*CVD) method reported in our previous studies (Figure 1).^54,61,62^ For the *im*CVD process, C/A-1° miscut sapphire substrates and usual C-cut sapphire substrates were used for *sc*-MoS_2_ and *ep*-MoS_2_, respectively. To elucidate the crystallographic characteristics of the synthesized *sc*-MoS_2_ and *ep*-MoS_2_ samples, standard single-crystal characterization techniques were employed.^52,53^ The initial examination involved categorizing grown MoS_2_ into partially grown (Figure 1a and e) and fully grown (Figure 1b and f) stages. For 5 min of growth on a C/A-1° miscut sapphire substrate, *sc*-MoS_2_ exhibited the formation of triangular, unidirectionally aligned 2H MoS_2_ monolayers, consistent with previously reported observations (Figure 1c).^11,24,25,52^ In contrast, partially grown *ep*-MoS_2_ on a C-cut sapphire substrate displayed an equivalent distribution of A and B orientation grains (Figure 1g). However, in the fully grown stage, the distinction between *sc*-MoS_2_ and *ep*-MoS_2_ became indiscernible via optical microscopy. An additional scanning electron microscopy (SEM) image of *sc*-MoS_2_ is shown in Figure S3. Scanning transmission electron microscopy (STEM) and selected area electron diffraction (SAED) analyses revealed the concurrent presence of A and B orientation domains within the *ep*-MoS_2_ samples (Figure 1h). In contrast, Figures 2 and 1d show consistent SAED patterns of *sc*-MoS_2_, indicating a uniform atomic orientation in the regions analyzed (Figure 1d).

**Figure 1:**
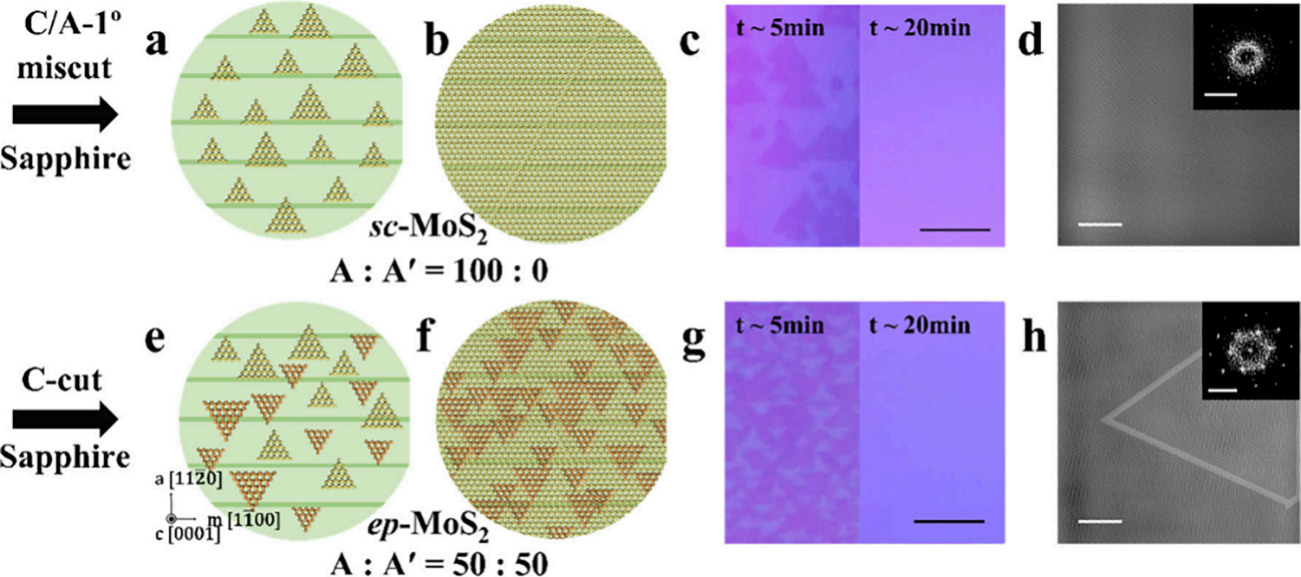
Friedel’s law violation in electron diffraction reveals pre-annealing-condition-driven crystal orientations in MoS_2_. Schematic illustration of epitaxially (a) partially grown and (b) fully grown *sc*-MoS_2_. Corresponding image by (c) OM and (d) inverse fast Fourier transform (IFFT) of TEM data of *sc*-MoS_2_, respectively. Schematic illustration of epitaxially (e) partially grown and (f) fully grown *eq*-MoS_2_. Corresponding image by (g) OM and (h) IFFT of TEM data of *eq*-MoS_2_, respectively. The scale bar is 10 μm for the OM image, 5 nm for the TEM image, and 5 nm^–1^ for the SAED pattern.

**Figure 2:**
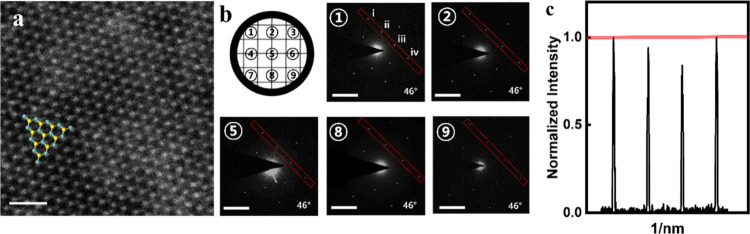
Verification of single crystallinity in the full-film *sc*-MoS_2_ by SAED analysis. (a) Atomic resolution HAADF–STEM image of monolayer 2H MoS_2_ in area 5 of the TEM grid. The scale bar is 1 nm. (b) SAED patterns acquired from different positions across the sample, as indicated by the grid overlay. The scale bar is 5 nm^–1^. (c) Corresponding line profile of the SAED intensity measured at area 5 on the TEM grid. A consistent intensity enhancement of the ii spots (A′ orientation) relative to the iii spots (A orientation) is observed across all measured locations. This uniformity confirms that the entire film is single-crystalline with a globally aligned orientation.

In particular, Figure 2a presents an atomic resolution high-angle annular dark-field STEM (HAADF–STEM) image of the 2H MoS_2_ monolayer acquired from area 5 of the TEM grid, clearly revealing a unidirectionally aligned MoS_2_ monolayer structure. SAED patterns were collected from nine distinct positions across the sample, as indicated by the grid overlay in Figure 2b. The corresponding SAED intensity line profiles for each position (Figure 2c and Figure S4c) consistently exhibit an intensity enhancement at the ii spots (B orientation) relative to that at the iii spots (A orientation) at all measured locations. Statistical validation of each normalized SAED intensity was conducted by *t* tests for the *sc*-MoS_2_ film (see the Supporting Information). This result provides strong evidence that synthesized MoS_2_ exclusively adopts the A orientation rather than a mixture of the A and B orientations. This uniformity, along with the additional SAED patterns from the remaining nine locations (Figure S4b), confirms the single-crystalline characteristics of the entire film with a globally aligned orientation. Furthermore, the atomic resolution HAADF–STEM images (Figure S5) also confirm that *sc*-MoS_2_ was grown in a unidirectional manner. Figure 3 shows the LEED patterns measured on large-area *sc*-MoS_2_ samples depending on the E-beam energy. While a 6-fold LEED pattern is observed at certain E-beam energy, obvious 3-fold LEED patterns are observed on *sc*-MoS_2_ (Figure 3a–c). However, 3-fold LEED patterns appear in an inverted configuration depending on the E-beam energy. On the contrary, *ep*-MoS_2_ exhibits a 6-fold LEED pattern, independent of the energy of the electron beam (Figure 3d–f). Although the influence of electron beam energy on this phenomenon will be discussed in detail later, these data already confirm that LEED patterns exhibiting 3-fold symmetry can effectively distinguish between A and B orientations. Technically, the structure factor (*F*_*g*_) for a monolayer MoS_2_, written for an incident electron wave, can differ from its inversion counterpart not only in amplitude but also in phase. Consider that the scattering amplitude in the kinematic sense is

1 $$F_{g}=\int \rho \left(r\right)\exp\left(-2\pi ig\cdot r\right)d^{3}r$$

where the term ρ­(***r***) effectively represents the electrostatic potential distribution of the 2D layer. When this distribution lacks inversion symmetry, i.e., ρ­(***r***) ≠ ρ­(−***r***), equality *F*_*g*_ = *F*_–*g*_ can no longer be assumed. While the resulting differences between *F*_*g*_ and *F*_–*g*_ might be subtle when probing the sample with high-energy X-rays (Figure 4a), especially under conditions minimizing multiple scattering, LEED presents a different scenario (Figure 4b). Under LEED conditions, electrons with kinetic energies in the tens of electron volts range exhibit strong dynamical interactions. Electrons, being charged particles, interact strongly with the Coulomb potential of the atomic nuclei and core electrons within the crystal. This interaction is mediated by the exchange of virtual photons, a fundamental process in quantum electrodynamics (QED). The lower kinetic energy of the electrons in LEED enhances the interaction cross section, leading to multiple scattering events. In contrast, X-rays, being electromagnetic waves, primarily interact with the electron density through Thomson scattering. The interaction cross section for Thomson scattering is significantly smaller than that for Coulomb scattering, resulting in weaker interactions and a dominance of single-scattering events. Therefore, X-rays, being uncharged, do not interact with the periodic potential in the same manner as charged particles,

**Figure 3:**
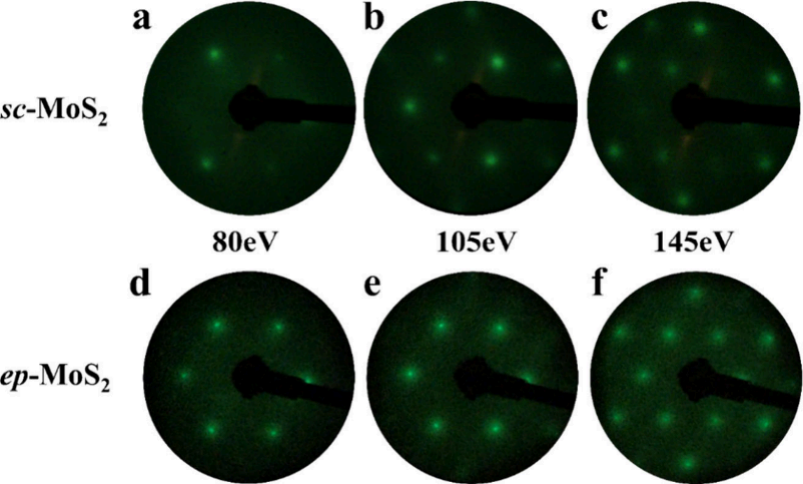
Energy-dependent LEED patterns of *sc*-MoS_2_ and *ep*-MoS_2_. Inversion of 3-fold symmetry enables crystal orientation distinction. LEED patterns obtained from a large-area (a–c) *sc*-MoS_2_ and (d–f) *ep*-MoS_2_ depending on the acceleration voltage, respectively. The full-film *sc*-MoS_2_ monolayer shows 3-fold LEED pattern in (a) 80 eV, (b) 100 eV, and (c) 140 eV, respectively, while the 6-fold LEED pattern is observed at same voltage of (d) 80 eV, (e) 100 eV, and (f) 140 eV, respectively. Notably, the 3-fold LEED patterns invert by increasing acceleration voltage, enabling effective distinction between the A and A′ orientations.

**Figure 4:**
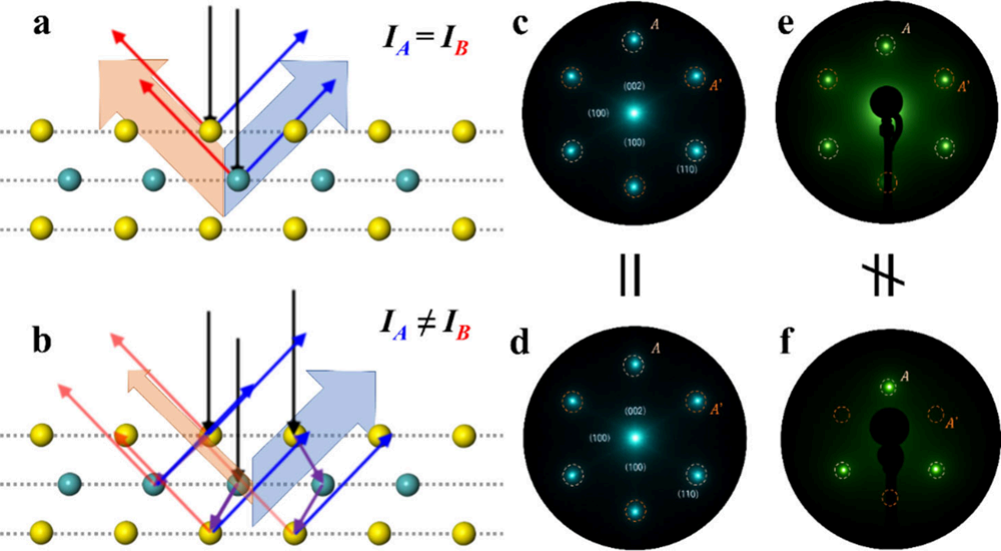
Friedel’s law violation in electron diffraction reveals the 2H MoS_2_ monolayer. (a) Schematic of radiation (X-ray) diffraction in the kinematic, elastic, single-scattering regime, where Friedel’s law holds and intensities of (*h*, *k*, *l*) and (−*h*, −*k*, −*l*) reflections are equal. (b) Schematic of electron diffraction from a 2H MoS_2_ monolayer under dynamical conditions, highlighting multiple scattering and atom-to-atom inelastic scattering that break Friedel’s law and produce intensity asymmetry in Friedel’s pairs. (c and d) Radiation (X-ray) diffraction patterns of (c) *eq*-MoS_2_ and (d) *sc*-MoS_2_; both obey Friedel’s law and thus cannot be distinguished by intensity asymmetry. (e and f) Electron diffraction patterns of (e) *eq*-MoS_2_ and (f) *sc*-MoS_2_, exhibiting Friedel’s pair intensity asymmetry due to dynamical/inelastic effects, enabling discrimination between the two and revealing the 2H MoS_2_ monolayer.

Therefore, uncharged X-rays do not interact with the periodic potential in the same manner as charged particles, which renders them relatively insensitive to subtle differences in surface registry, stacking, or domain orientation. Consequently, the diffraction patterns of *sc*-MoS_2_ and *eq*-MoS_2_ are largely indistinguishable under X-ray measurements (Figure 4c and d). By contrast, in LEED, where low-energy electrons interact relatively strongly with the surface periodic potential and undergo significant dynamical scattering, fine differences in symmetry and stacking give rise to distinct diffraction signatures, leading to repeated scattering channels, where each “step” in the reciprocal space modifies the amplitude according to the product of the structure factors and the phase factors. If the structure lacks inversion symmetry, these multistep scattering processes do not cancel out in a way that would equalize the intensities of ***g*** vs −***g***. Consequently, the difference in intensities can become significantly amplified in LEED, sometimes to the point of being visually apparent in the diffraction pattern as a noticeable imbalance among nominally symmetry-related spots (Figure 4e and f). An additional point is that, in a monolayer, the potential the electrons experience is strongly two-dimensional. The reflection geometry of LEED further means that the electron wave may partially reflect from the underlying substrate interface or vacuum boundary, introducing interference that can sharpen or enhance any existing asymmetry. Therefore, in a monolayer of *sc*-MoS_2_, distinct, non-equivalent intensities can be seen for, say, (1, 0) and (1, 0) in the LEED pattern at specific energies (Figure S7).

It is important to contrast LEED with electron diffraction in TEM, because the markedly different electron energies and scattering geometries give rise to distinct multiple-scattering characteristics and differential sensitivities to inversion symmetry. During TEM measurements, a high-energy electron beam of about 60–300 keV passes through the sample. Even with an extremely thin specimen, such as monolayer MoS_2_, strong interactions with the crystal lattice can induce multiple scattering, making diffraction intensities dependent on both the magnitudes and phases of the structure factors. However, the ultrathin thickness of monolayer MoS_2_ at around 0.65 nm can limit the extent to which these dynamical effects fully evolve in TEM.

Although recent studies have reported 3-fold LEED patterns as evidence of unidirectional growth, systematic investigations into this phenomenon have been insufficient.^11,23–25,27–29,50,63^ For instance, numerous studies lack details on the precise E-beam energy value or do not clarify which diffraction spot along the ***k*** or ***k′*** direction exhibits the stronger intensity. Practically, this LEED-based discrimination is valuable to verifying the structural quality and orientation of the sample. If a monolayer MoS_2_ region shows a ring-shaped distribution of diffraction, it usually originates from a polycrystalline structure. Alternatively, a 6-fold symmetry with no obvious intensity mismatch is more consistent with two overlapping mirror-symmetric sublattices. In contrast, a 3-fold or asymmetrical arrangement of intensities has been suggested as evidence that the domain is unidirectionally oriented. However, we again emphasize that this merely reflects an imbalance between the populations of A and B domains rather than indicating a perfectly unidirectional orientation. To provide detailed information to demonstrate the unidirectionality of MoS_2_ with LEED analysis, we extracted LEED *I*–*V* curves for each of these main points by systematically measuring LEED images over a range of electron beam energies (for example, 50–200 eV) (Figure 5a). All LEED patterns used to extract *I*–*V* curves are shown in Figures S7 and S8. This method offers insight into how the electronic wave interacts with the topmost atomic plane under varying conditions of dynamical scattering. Indeed, the amplitude and phase of the structure factors responsible for each of these spots will shift as the electron wavelength changes, leading to distinct maxima and minima in the intensity profiles.

**Figure 5:**
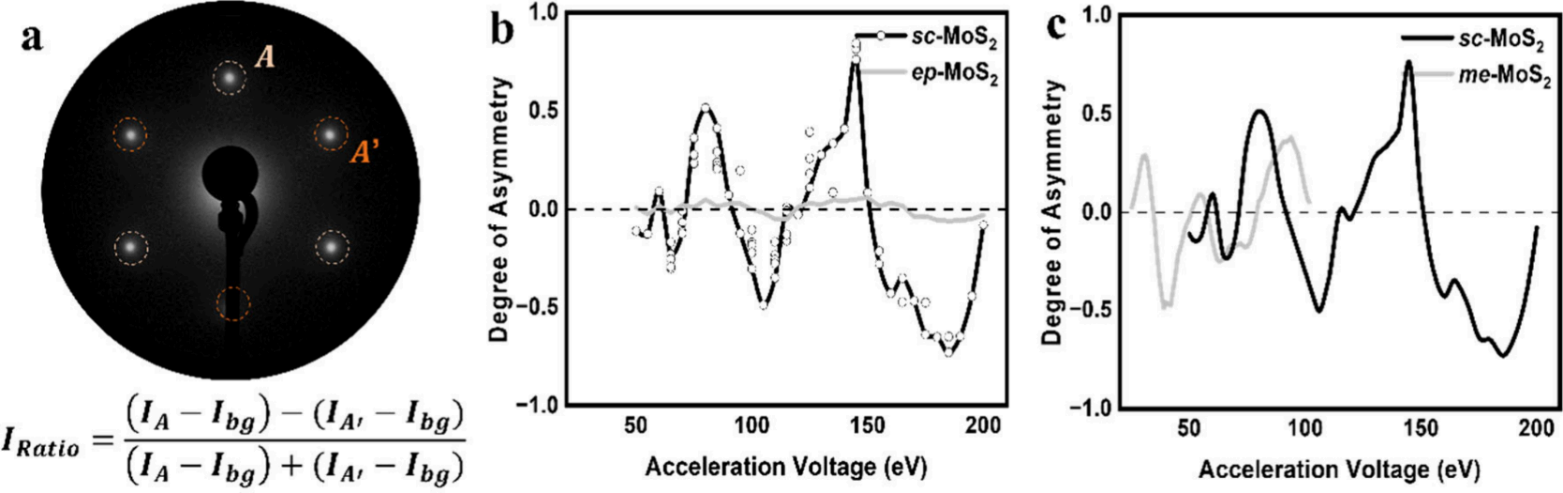
Characterization of *sc*-MoS_2_ and *ep*-MoS_2_ monolayers via the asymmetry degree of LEED patterns varying the acceleration voltage. (a) LEED *I*_ratio_ (degree of asymmetry) was calculated based on the net intensities, obtained by subtracting the background from the absolute brightness of each main spot (A′ and A). (b) Degrees of asymmetry for *sc*-MoS_2_ and *ep*-MoS_2_ samples were plotted against the acceleration voltage. *I*_ratio_ of the *sc*-MoS_2_ sample exhibited an extremum at specific voltages, whereas *I*_ratio_ for the *ep*-MoS_2_ sample converged to zero. (c) *I*_ratio_ of the *sc*-MoS_2_ sample was compared to both experimental reference data [from mechanically exfoliated monolayer MoS_2_ on SiO_2_ (300 nm)/Si].^64^ The referenced study reports measurements only in the 20–100 eV range, with no data available above 100 eV.

In Figure 5b, *sc*-MoS_2_ exhibited extrema in the intensity ratio at 60, 65, 80, 105, 145, and 185 eV. In contrast, the intensity ratio of *eq*-MoS_2_ converged to zero across the entire energy range. The significant intensity difference between A­(***g***) and A′(−***g***) spots in *sc*-MoS_2_ but not in the *eq*-MoS_2_ sample is a direct manifestation of the inelastic scattering resulting from broken inversion symmetry and the three-layered structure of the 2H MoS_2_ monolayer, amplified by the dynamical scattering conditions inherent in LEED. The origin of this asymmetry can be understood through a dynamic scattering model of electron diffraction. The key factor differentiating *sc*-MoS_2_ from *eq*-MoS_2_ lies in the presence (or absence) of long-range crystalline order and inversion symmetry. In *sc*-MoS_2_, the 2H polytype structure, consisting of three atomic layers stacked in a specific S–Mo–S sequence, lacks inversion symmetry. This asymmetry manifests in *F*_*g*_, which, for an incident electron wave, can differ from its *F*_–*g*_ not only in amplitude but also in phase, where ρ­(***r***) = ρ­(−***r***) is effectively the electrostatic potential distribution of the 2D sheet and when ρ­(***r***) one can no longer assert *F*_*g*_ = *F*_–*g*_ (eq 1). As previously mentioned, the distinct interaction mechanisms of electrons and X-rays with the crystal lattice lead to enhanced multiple scattering in LEED. Under LEED conditions, electrons exhibit strong dynamical interactions, and this leads to repeated scattering channels.

For comparison, Figure 5c plots our data alongside *I*–*V* curves obtained from μ-LEED measurements on mechanically exfoliated MoS_2_ (*me*-MoS_2_) on a SiO_2_ substrate, which are the previously reported μ-LEED data^64^ for comparison. While both data sets exhibit similar overall trends, a noticeable shift in the patterns is observed. This shift can be attributed to strain effects arising from substrate interactions in epitaxially grown MoS_2_ on sapphire, which are absent in *me*-MoS_2_ on SiO_2_.^65,66^ Moreover, in the case of μ-LEED, the grain orientation is not uniquely defined and the distinction between A and B orientations was made without accounting for substrate effects. Consequently, our results provide more reliable and standard reference data for analyzing *sc*-MoS_2_ films epitaxially grown on sapphire substrates.

These findings can be rationalized within the framework of the dynamic scattering theory. In *sc*-MoS_2_, the absence of inversion symmetry amplifies multistep scattering effects, producing clear intensity asymmetries between nominally equivalent diffraction spots and giving rise to resonance-like variations at specific electron energies. By contrast, *eq*-MoS_2_ lacks long-range crystalline order, and the random orientation of its constituent flakes averages out such asymmetries. As a result, the intensity ratio converges toward zero, reflecting both structural disorder and enhanced inelastic scattering. Thus, the pronounced energy-dependent asymmetry in *sc*-MoS_2_ and its absence in *eq*-MoS_2_ provide a direct structural fingerprint that distinguishes truly single-crystalline films from their polycrystalline or exfoliated counterparts.

In summary, the non-centrosymmetric characteristic of monolayer MoS_2_ explains why one can see little to no Friedel law violation in conventional X-ray diffraction yet observe distinct intensity differences in electron diffraction techniques. We explore the LEED patterns of unidirectionally grown single-crystal monolayer MoS_2_, focusing on the characteristic 3-fold diffraction features that become evident at various incident electron energies. By acquiring a series of LEED images from 50 to 200 eV and extracting the intensities of key diffraction spots, we construct *I*–*V* curves that clearly reveal the dynamic scattering resonances associated with the topmost layer of MoS_2_. We demonstrate that the three main spots, spaced by 120°, show distinct or asymmetric intensity variations, confirming both the absence of inversion symmetry in the monolayer and the unidirectional alignment of the triangular domains. These findings suggest that LEED *I*–*V* analysis can serve as a robust diagnostic tool to determine monolayer orientation, confirm single-crystal behavior, and potentially refine structural parameters for monolayer TMDs. The observations presented here fill a gap in the literature, where prior discussions of 3-fold LEED in MoS_2_ were mostly qualitative and did not systematically link the energy-dependent evolutions of intensity to the underlying lattice asymmetry and growth directionality. The synergy of non-centrosymmetric structure factors and multiple scattering thus provides a clear demonstration of inversion symmetry breaking at the atomic scale, highlighting why LEED is such a powerful tool for analyzing monolayer TMD surfaces and confirming unidirectionally grown crystals. Overall, our results may serve as a guideline for applying LEED to the analysis of single-crystalline, non-centrosymmetric 2D materials beyond MoS_2_.

## Supplementary Material



## Abbreviations Used

CVD: chemical vapor deposition
*F*_*g*_: structure factor
HAADF–STEM: high-angle annular dark-field scanning transmission electron microscopy
*I*–*V*: current–voltage
IMCVD: inorganic molecular chemical vapor deposition
LEED: low-energy electron diffraction
MoOCl_4_: molybdenum­(IV) oxychloride
MoS_2_: molybdenum disulfide
MoSe_2_: molybdenum diselenide
PL: photoluminescence
SAED: selected area electron diffraction
*sc*-MoS_2_: single-crystal MoS_2_
*ep*-MoS_2_: epitaxially grown MoS_2_ with nearly equal populations of A and A′ orientations
SHG: second harmonic generation
TEM: transmission electron microscopy
TMD: transition metal dichalcogenide
WSe_2_: tungsten diselenide
WS_2_: tungsten disulfide
