# Supplementary material for: Revisiting Dynamical Theory To Elucidate Friedel’s Law Breaking in Low-Energy Electron Diffraction as Strong Evidence of Unidirectional Growth of Monolayer 2H MoS2
Source: Nano Lett. 2026 Jan 9;26(2):747–54. doi: 10.1021/acs.nanolett.5c05097 (PMC12833839; doi:10.1021/acs.nanolett.5c05097)
Supplement: Supplementary file 1 [file nl5c05097_si_001.pdf]

Supporting Information

Revisiting Dynamical Theory to Elucidate Friedel's Law Breaking in Low-Energy Electron Diffraction as Strong Evidence of Unidirectional Growth of Monolayer 2H MoS_2

Dohoon Kim,[†] *Joohee Oh,*[†,‡] *Chaehyeon Ahn,*[$] *Joonbyeong Jeon,*[†] *Hyeeree Joo*[†] *and Hyunseob Lim*[†,‡,$,‖,*]

[†]Department of Chemistry, Gwangju Institute of Science and Technology (GIST), 123 Cheomdangwagi-ro, Buk-gu, Gwangju 61005, Republic of Korea

[‡]Center for Quantum Conversion Research, Institute for Basic Science (IBS), Gwangju, 61005, Republic of Korea

[$]GIST InnoCORE AI-Nano Convergence Institute for Early Detection of Neurodegenerative Diseases, Gwangju Institute of Science and Technology (GIST), 123 Cheomdangwagi-ro, Buk-gu, Gwangju 61005, Republic of Korea

[‖]Department of Semiconductor Engineering, Gwangju Institute of Science and Technology (GIST), 123 Cheomdangwagi-ro, Buk-gu, Gwangju 61005, Republic of Korea

E-mail: hslim17@gist.ac.kr

Experimental Section

Materials

sulfur powder (99.98%), Molybdenum (IV) oxychloride ($MoOCl_4$, 97.0%), sulfur (S, sublimed, 99.5%) powder, sodium hydroxide (NaOH, bead, 98.0%), acetone (99.5%), isopropyl alcohol (94.5%), deionized water (99.999%), N_2 gas (99.999%), Ar gas (99.999%), polystyrene (PS, 350,000 g/mol) and toluene were used in this experiment.

Preparation of Monolayer 2H-MoS_2 films

Monolayer 2H-MoS_2 films were synthesized on C/A-1° miscut and C-cut sapphire substrates using an inorganic molecular chemical vapor deposition (*im*CVD) method.[1, 2] Prior to synthesis, the substrates were sequentially rinsed for 30 seconds each in acetone, isopropyl alcohol, and deionized water, followed by N_2 gas blowing to remove any surface contaminants. The synthesis process consisted of a two-step procedure: a substrate pre-annealing step followed by a growth step (**Figure S1**). The growth conditions were identical for both *sc*-MoS_2 and *ep*-MoS_2 films except the substrate type.[3] During the pre-annealing step, the furnace was ramped from room temperature to 1100°C over 40 minutes (at a rate of 26.9°C/min). To obtain *sc*-MoS_2, the sapphire substrate was pre-annealed at 1100°C for 2 hours under a flow of Ar (80 sccm) and O_2 (20 sccm) by a mass flow controller (MFC). To obtain *ep*-MoS_2, the substrate was pre-annealed under identical temperature and time conditions but using a C-cut sapphire substrate. Following the pre-annealing, the growth step was conducted at 950°C for 20 minutes. $MoOCl_4$ served as the inorganic molecular precursor, with sulfur powder as a chalcogen source and NaOH as a catalyst, respectively (**Figure S2**).[4, 5] During growth, high-purity Ar was used as the carrier gas at a flow rate of 80 sccm, regulated by a MFC. The $MoOCl_4$ precursor, housed in a stainless-steel chamber heated by a heating

band, was maintained at 70°C and supplied at a constant pressure of 2.0×10^{-4} Torr using a metering valve. Sulfur powder (1000 mg) was placed in an alumina boat and heated to 120°C. Further details can be found in our previously published work.[1] Within the three-zone furnace, the sapphire substrate was positioned at the center of the high-temperature zone (Zone III). The sulfur source was placed 45 cm upstream in the center of Zone I, and the NaOH catalyst was positioned 15 cm downstream from the sulfur source. After the 20 minutes growth period, the furnace was slid away from the sample to ensure rapid cooling to room temperature, finalizing the synthesis.

Transfer Method

To transfer MoS_2, a PS film was utilized. 9.49 g of PS (350,000 g/mol) was dissolved in 131 ml of toluene, and PS solution was spin coated onto the MoS_2/Sapphire with 1000/4000 rpm for 10/40 seconds, respectively. PS/MoS_2 was immersed in DI for 10 minutes and transfer onto the Quantifoil TEM grid.

LEED Characterization and Correction Process

To ensure reliable and accurate Low-energy electron diffraction (LEED) intensity-voltage (I-V) measurements, a systematic data acquisition and correction protocol was employed.[6, 7] First, the MoS_2 surface was cleaned by thermal annealing process for 24 hours to get rid of organic particle on MoS_2 surface, by applying 4.0 V and 3.26 A to the tungsten filament located under the sample stage, to which sample bias of 1 kV was applied. The surface temperature of sample was maintained at 430°C during the annealing process. Following the cleaning process, LEED patterns were acquired in an ultra-high vacuum (UHV) condition below 4.0×10^{-10} Torr, using a constant incident beam current of 1.00 μA. This pressure is significantly lower than the typically recommended pressure of 5.0×10^{-10} Torr for LEED

measurements, indicating exceptionally clean surface conditions ideal for high-quality LEED pattern acquisition.[8] The raw underwent a series of processing steps to ensure accuracy. First, to minimize measurement artifacts and improve statistical reliability, the intensities of six symmetry-equivalent spots were measured and averaged for each diffraction order. Second, a deblurring process was applied to sharpen the peaks and improve energy resolution. Process was conducted by subtracting the background intensity (I_{bg}) from the raw intensity of each diffraction spot (I_A and I_B). Subsequently, a correction for the inner potential was applied to align the acceleration energy.[9-12] Finally, to quantify the structural asymmetry, the degree of asymmetry (I_{Ratio}) was calculated from the corrected net intensities of a pair of non-equivalent spots (**A** and **B**). The ratio was determined using the following formula:

$$I_{Ratio} = \frac{(I_A - I_{bg}) - (I_B - I_{bg})}{(I_A - I_{bg}) + (I_B - I_{bg})}$$

where I_A and I_{B} represent the corrected intensities of the respective diffraction spots, and I_{bg} is the averaged background intensity.

Statistical Validation (t-tests) of Normalized SAED Intensities (i–iv) for *sc*-MoS_2[13]

This Supplementary Note reports a statistical evaluation of the normalized SAED spot intensities corresponding to **Figure 2c** for a monolayer 2H-MoS_2 single crystal. Intensity of spot i was used as the internal normalization anchor ($I_i(n) = 1.0$). Two-tailed one-sample t-tests were performed against $\mu = 1.0$ for spots ii–iv, and pairwise Welch t-tests (unequal variances) were used to compare spots. Significance was set to $\alpha = 0.05$ (Holm corrected for multiple comparisons). Nine SAED patterns for measure on different regions were used for statistical analysis.

t-test results: Mean ± SD values were: ii = 0.958 ± 0.021, iii = 0.808 ± 0.058, and iv = 0.996 ± 0.0068. Relative to unity, ii was slightly but significantly lower ($t = -6.09$, $p = 3.0\times10^{-4}$; 95% CI 0.943–0.974), iii was markedly lower ($t = -9.89$, $p < 1\times10^{-5}$; 95% CI 0.763–0.853), while iv was statistically indistinguishable from 1.0 ($t = -1.58$, $p = 0.15$; 95% CI 0.991–1.002). Pairwise tests confirmed clearly separated distributions among the spots: ii vs iii ($t \approx 7.31$, $p < 1\times10^{-5}$), ii vs iv ($t \approx -5.28$, $p = 4.0\times10^{-4}$), and iii vs iv ($t \approx -9.64$, $p < 1\times10^{-5}$). As expected for the normalization anchor, i and iv did not differ ($p = 0.15$), whereas i differed from ii and iii.

These statistics quantitatively support the trend observed in **Figure 2c**: regions (i) and (iv) exhibit nearly identical intensities, region (ii) is slightly lower, and region (iii) shows the weakest intensity, resulting in well-separated intensity clusters (i ≈ iv > ii > iii). The measured intensity ratios and absolute values are in excellent agreement with reported reference data for monolayer 2H-MoS_2 SAED spot intensities,[14] providing strong validation of our measurements. For transparency and reproducibility, the normalized spot intensity values used in **Figure 2c** are listed below.

Normalized intensity values for 9 patterns

i: [1, 1, 1, 1, 1, 1, 1, 1, 1]

ii: [0.99149, 0.92919, 0.95502, 0.93680, 0.94051, 0.96005, 0.97877, 0.96174, 0.97215]

iii: [0.82078, 0.74166, 0.72919, 0.73682, 0.84083, 0.83248, 0.88830, 0.86230, 0.82078]

iv: [1, 1, 0.99880, 0.98394, 0.98495, 1, 1, 1, 1]

Characterizations

The optical images were obtained using an optical microscope (BX53MRF-S; Olympus). The Bright-field TEM images and the SAED pattern were obtained at high resolutions using a

high-resolution transmission electron microscope (JEOL, JEM-2100 LaB6) at an accelerating voltage of 200 kV. HAADF-STEM images were obtained at atomic resolutions using a high resolution double cs-corrected transmission electron microscope (JEOL, JEM-ARM300F2) at an accelerating voltage of 80 kV on Holey Carbon Coated TEM Grid. LEED patterns were obtained at accelerating voltages of 20–200 eV, in constant beam-current mode (1.00 μA) using a LEED–AES spectrometer (Vacuum Microengineering, BDL800IR). The Raman (mapping) and PL (mapping) measurements were performed by a lab-made 532 nm green laser. The XPS analysis was performed using the NEXSA system (Thermo Fisher Scientific).

LEED Calculation and condition

The calculation is performed using the AQuaLEED package,[15] which is based on the Barbieri/Van Hove SATLEED package.[16-18] The simulation modeled a single-crystal MoS_2 surface without considering sapphire substrate. The atomic positions for the MoS_2 monolayer model were defined in Cartesian coordinates, with the Mo atom located at (0, 0, 1.613) Å and the top S atoms and bottom S atoms at (a, $\frac{a}{\sqrt{3}}$, 3.226) Å and (a, $\frac{a}{\sqrt{3}}$, 0) Å, respectively. $a = 3.12$ Å. The structural parameters were defined with $p3m1$ symmetry (PatternSym = 3m), and the calculations were performed at a temperature of 320 K. The Debye temperature was set to 603 K, and the inner potential (optical potential) parameters were set to default values (MTConst = 6, Attenuation = 6).

Figures and Table

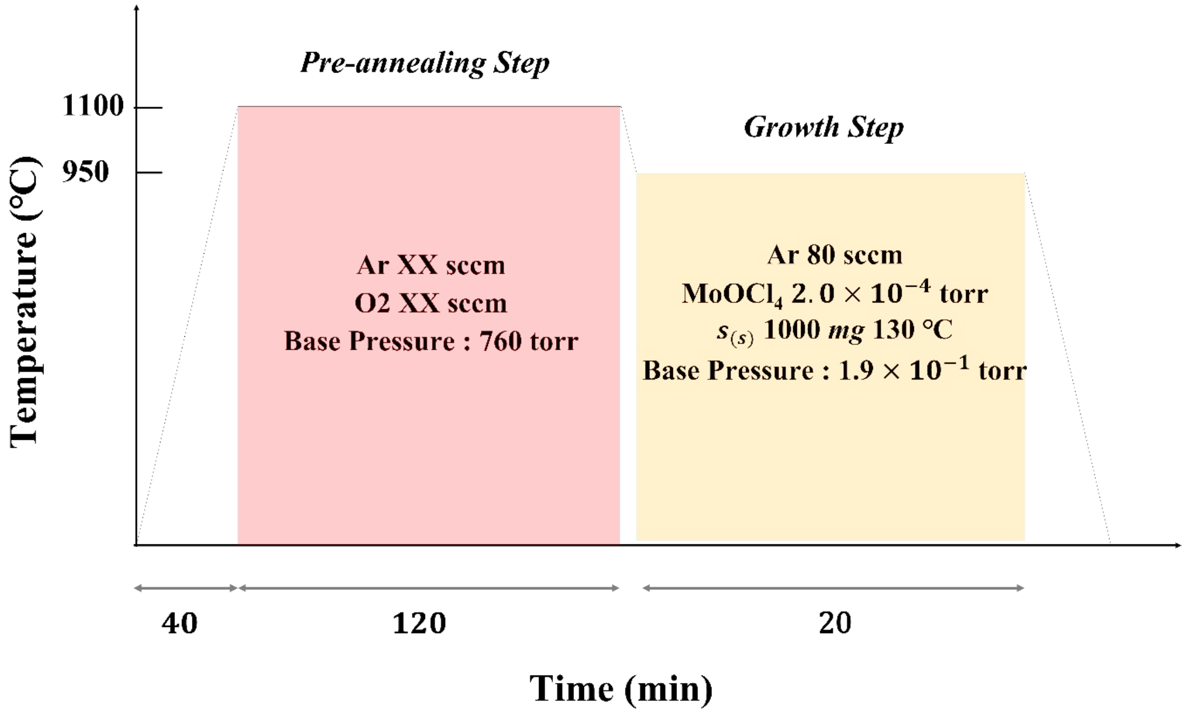

Figure S1. Schematic illustration of *im*CVD for experimental process of 2H-MoS_2 monolayer growth. The process is divided into two main stages. (Left, red box) The pre-annealing stage is conducted under an Ar/O_2 gas mixture with specific, unlisted flow rates at a base pressure of 760 torr (ambient pressure). (Right, yellow box) The growth stage is performed at a base pressure of 1.9×10^{-1} torr with an Ar carrier gas flow of 80 sccm, a $MoOCl_4$ precursor pressure of 2.0×10^{-4} torr, and a solid sulfur (S) source (1000 mg) heated to 130˚C.

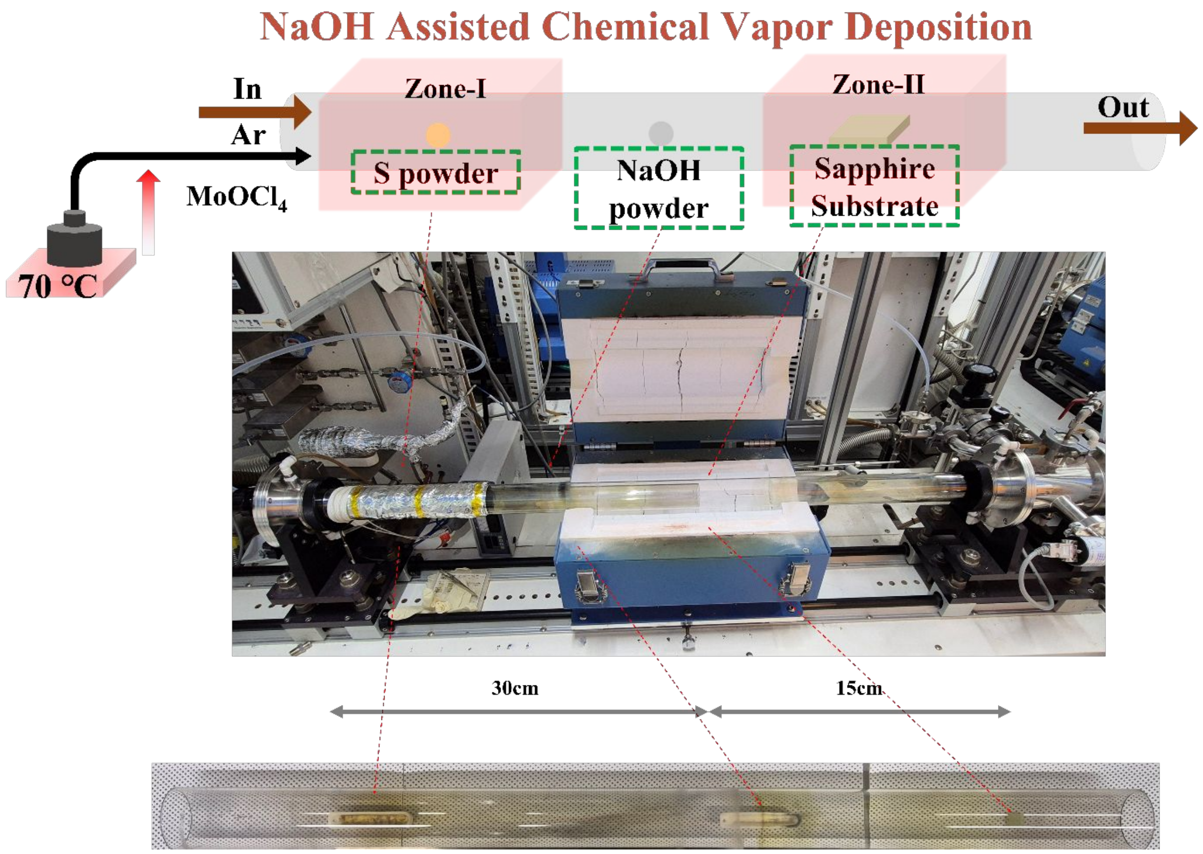

Figure S2. Schematic diagram and photographs of *im*CVD setup for *sc*-MoS_2 and *ep*-MoS_2 growth on sapphire with sulfur powders and $MoOCl_4$ as the precursors. The temperatures of sulfur and $MoOCl_4$ powders are set to be ~130 and ~70°C, respectively.

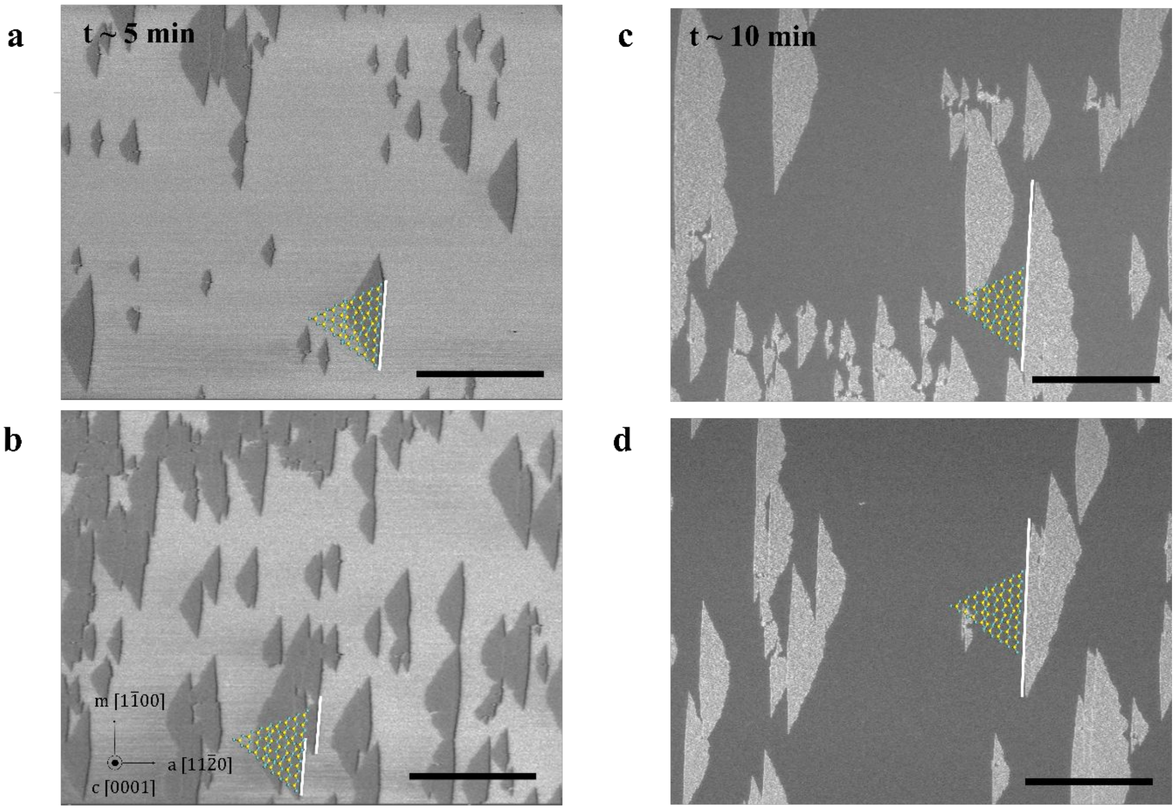

Figure S3. SEM images for as-grown unidirectional 2H-MoS_2 monolayer domains as a function of growth time. SEM images illustrating the morphological evolution of single-crystalline 2H-MoS_2 domains on a C/A miscut sapphire substrate with varying growth times. The images reveal the progressive growth and coalescence of the monolayer domains, demonstrating the temporal dependence of the film morphology. Indicating that (a) and (b) shows 2H-MoS_2 domains grown for approximately 5 minutes, and (c) and (d) shows different region of the sample grown for approximately 10 minutes. Scale bar is 20 μm.

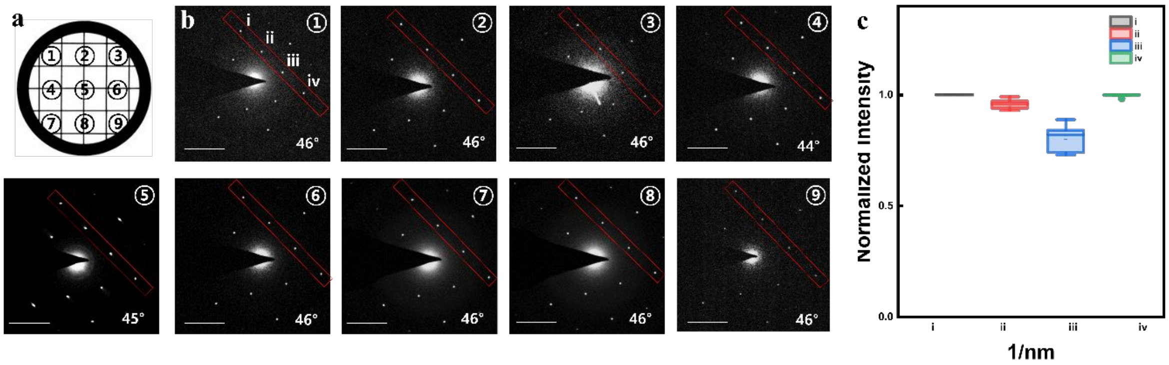

Figure S4. Verification of unidirectional orientation of full-film *sc*-MoS_2 by SAED pattern. (a) Schematic Figure for position of nine different position in TEM grid. (b) SAED patterns acquired from nine different positions across the sample, as indicated by the grid overlay. Scale bar is 5 nm^{-1}. (c) Corresponding line profiles of the SAED intensity measured at each nine spot on different position of TEM grid. A consistent intensity enhancement of the ii spots (**B** orientation) relative to the iii spots (**A** orientation) is observed across all measured locations. This uniformity confirms that the entire film is single-crystalline with a globally aligned orientation.

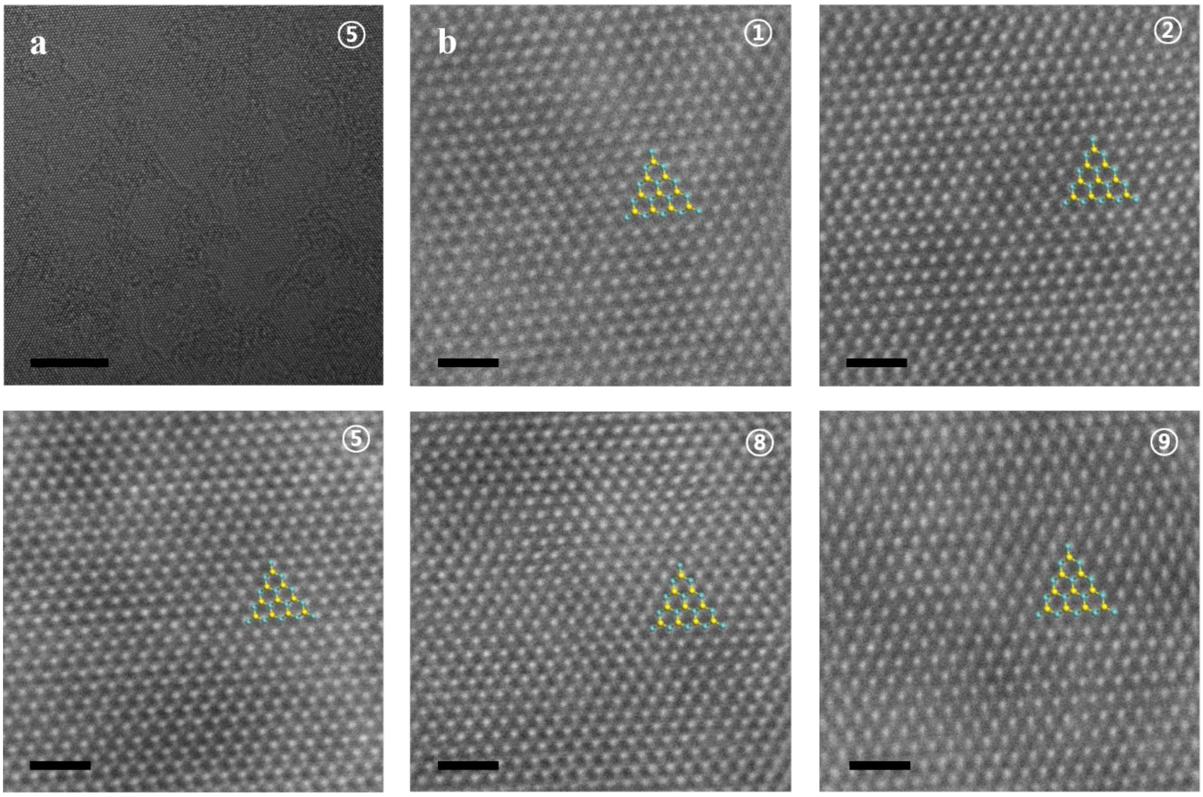

Figure S5. Verification of unidirectional orientation of full-film *sc*-MoS_2 by TEM image. (a) HAADF-STEM image for 2H-MoS_2 monolayer. Scale bar is 10 nm. (b) atomic resolution HAADF-STEM image for 2H-MoS_2 monolayer of *sc*-MoS_2 sample for different area in TEM grid (area 1, 2, 5, 8, 9, respectively). Scale bar is 1 nm. The *sc*-MoS_2 sample shows homogeneous texture and high crystallinity across the entire grid area.

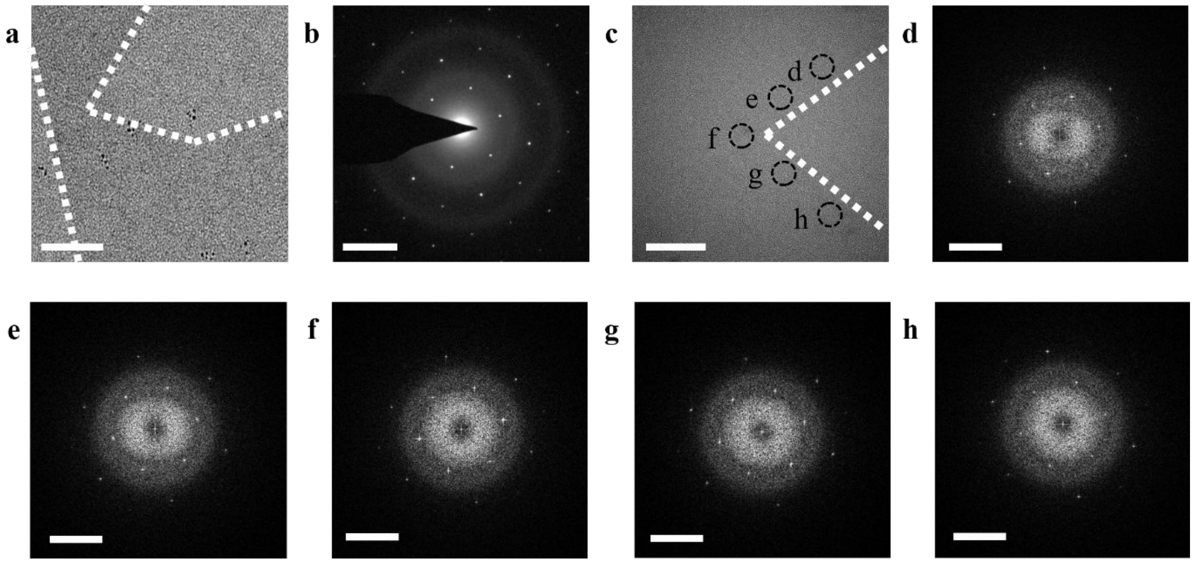

Figure S6. Verification of seamless stitching and uniform orientation in a single-crystalline 2H-MoS_2 monolayer. (a) Transmission electron microscopy (HR-TEM) image showing a stitched grain boundary region, scale bar is 80 nm. (b) Selected-area electron diffraction (SAED) pattern obtained from (a), confirming the boundary-free single-crystalline nature of the film, scale bar is 5 nm^{-1}. (c) A magnified HR-TEM image of merged boundary between grain and another grain, revealing a perfectly seamless stitched atomic lattice, scale bar is 20 nm. (d–h) Fast Fourier transform (FFT) patterns derived from five distinct locations within the imaged area, scale bar is 5 nm^{-1}. All five patterns exhibit identical hexagonal symmetry and orientation, which confirms the uniformly oriented, single-crystalline monolayer MoS_2 across the monolayer without observable grain boundaries.

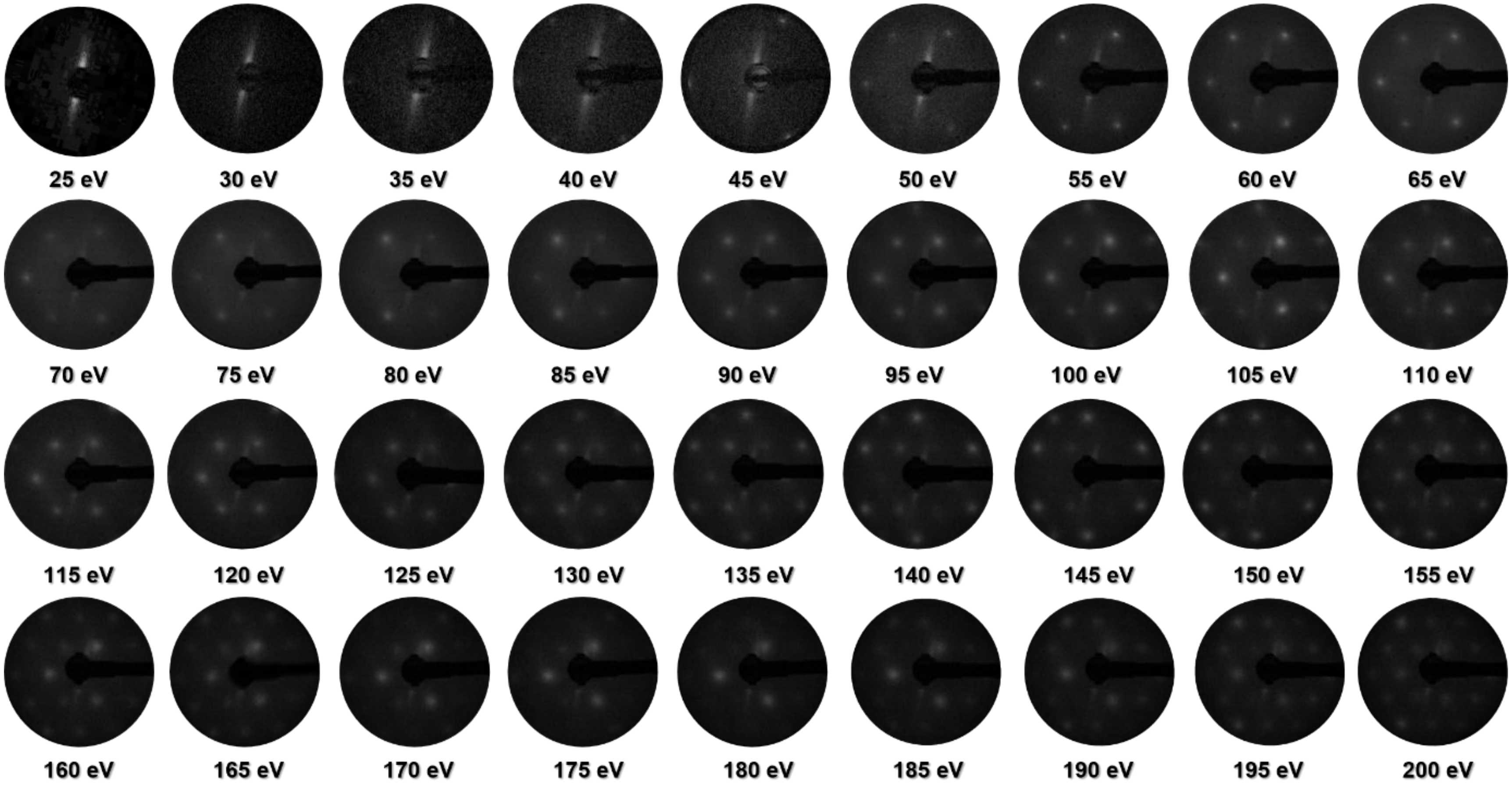

Figure S7. LEED I-V profiling images with unidirectional grown full film 2H-MoS_2 monolayer. The profiles were acquired under a constant beam current of 1.00 μA in the energy range of 50–200 eV. Data below 45 eV were excluded due to Instrumental limitations; No LEED patterns

could be captured from 25–35 eV because the large diffraction angles by low kinetic energies. Only few partial patterns were visible between 35–45 eV, and this data was ignored because the beam current was below the required 1.00 μA.

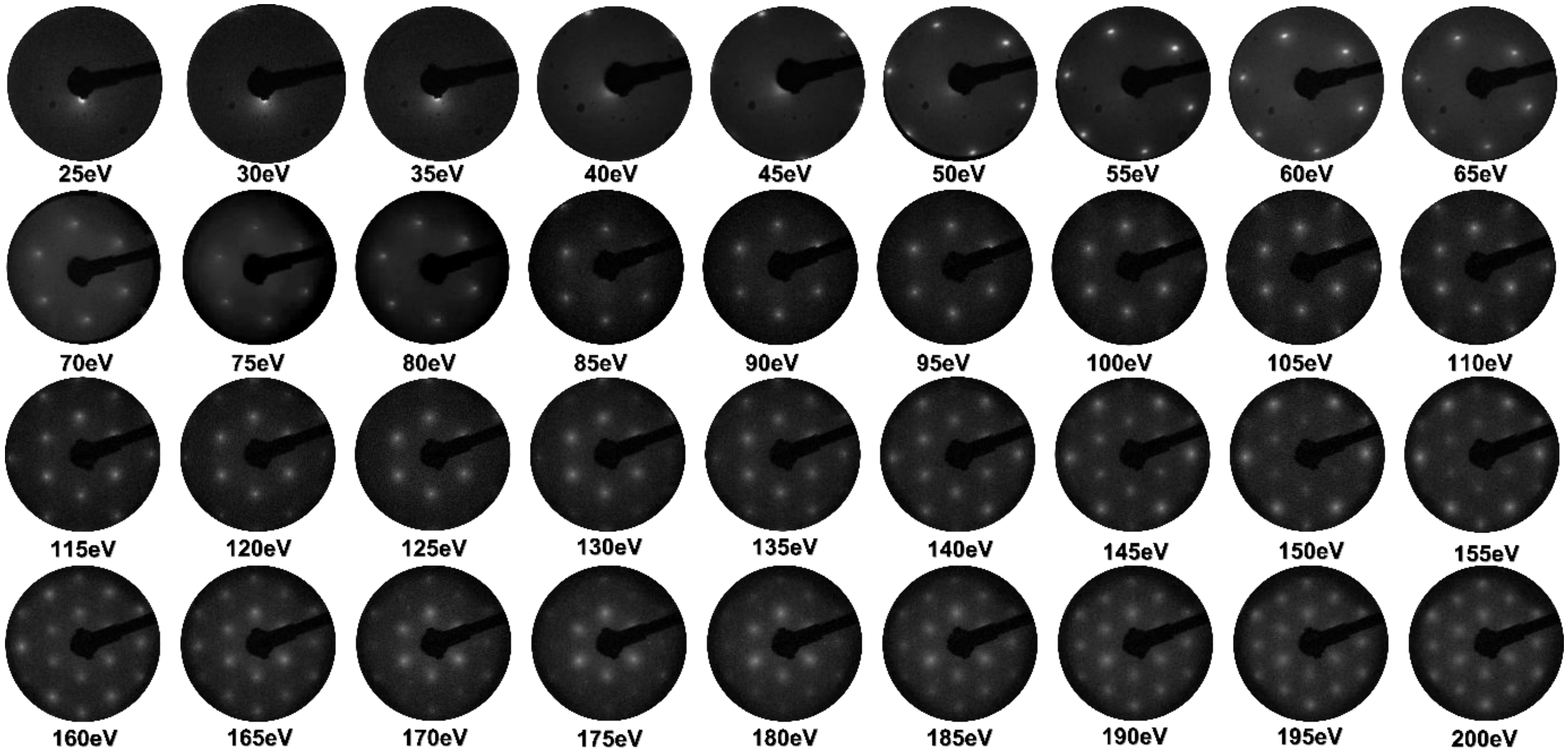

Figure S8. LEED I-V profiling images with equally grown full film 2H-MoS_2 monolayer. The profiles were acquired under a constant beam current of 1.00 μA in the energy range of 50–200 eV. Data below 45 eV were excluded due to Instrumental limitations; No LEED patterns could be captured from 25–35 eV because the large diffraction angles by low kinetic energies. Only few partial patterns were visible between 35–45 eV, and this data was ignored because the beam current was below the required 1.00 μA.

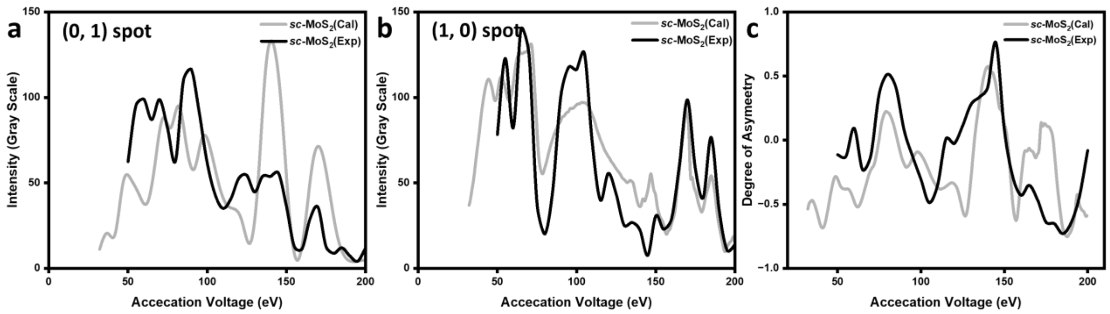

Figure S9. Calculated and experimental LEED I-V data of (a) (0, 1) spot, (b) (1, 0) spot, and (c) degree of asymmetry of *sc*-MoS_2 monolayer on sapphire, respectively.[15]

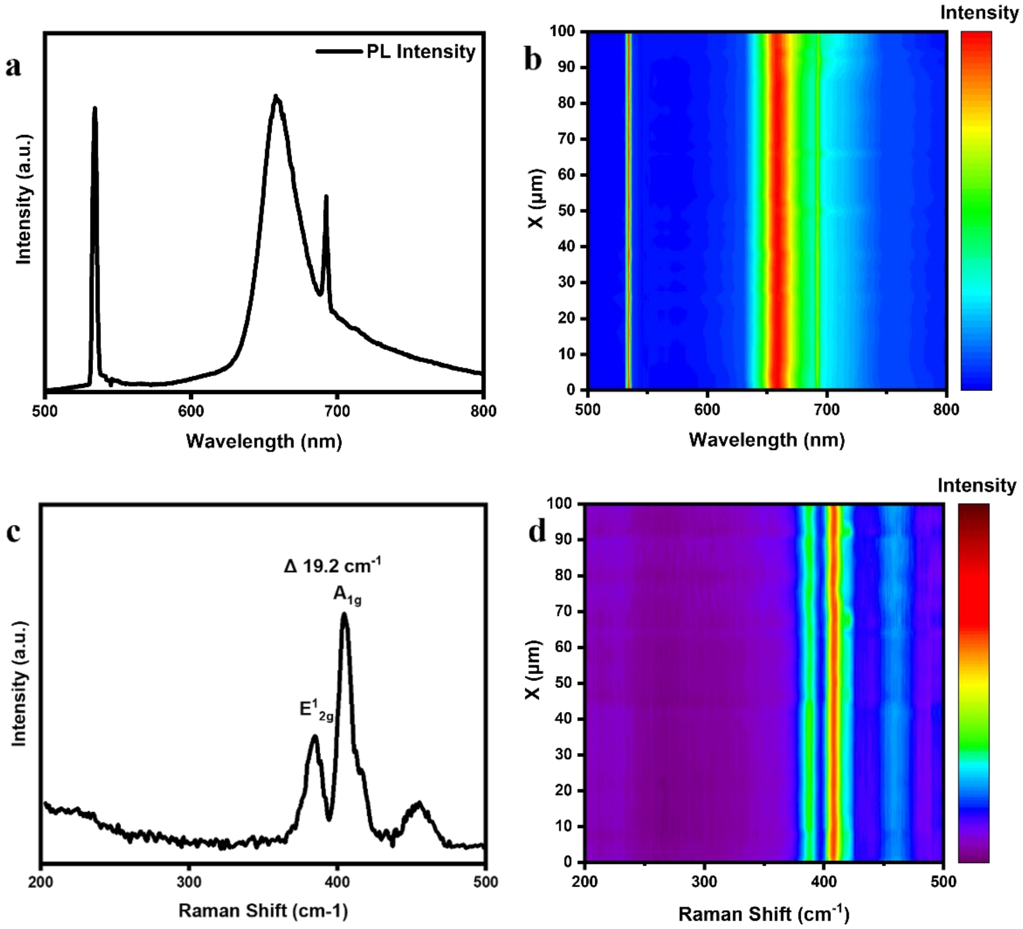

Figure S10. Characterization of the monolayer *sc*-MoS_2 film. (a) PL spectrum with an estimated FWHM of 68 meV. (b) PL line profile indicating high uniformity over a 100 µm. (c) Raman spectrum showing sharp E_{2g}^{1} and A_{1g} peaks at 385.0 and 404.2 cm^{-1}, respectively ($\Delta \approx$ 19.2 cm^{-1}), confirming the monolayer, and also along with the sharp peaks, establishes the high crystallinity of *sc*-MoS_2. Raman spectrum revealing the absence of the defect-induced LA mode at 226 cm^{-1}, indicative of high crystallinity. (d) Raman line profile showing high uniformity over a 100 µm.

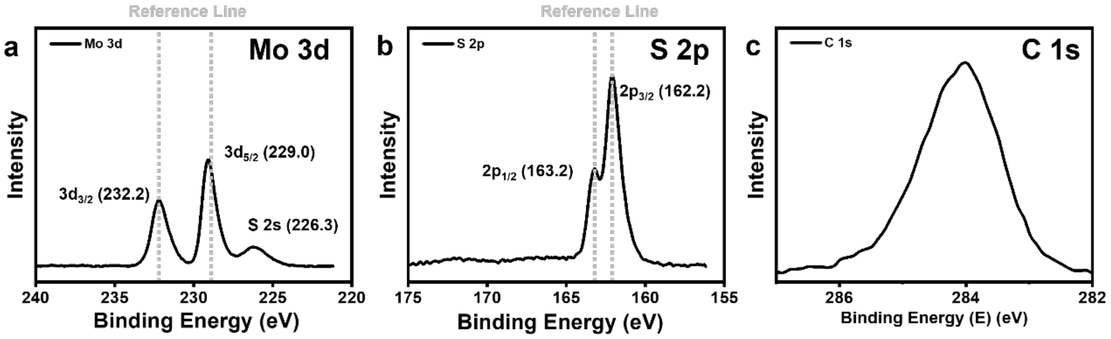

Figure S11. XPS spectrum for *sc*-MoS_2 film on Sapphire. (a) Mo 3d, (b) S 2p, and (c) C 1s core level spectra of single crystalline 2H-MoS_2 monolayer on sapphire substrate. The highly symmetric Mo 3d and S 2p peaks remain constant in position against reference data, closely matching intrinsic 2H-MoS_2. The Mo and S peak support the film's chemical purity and lack of doping effect.[19]

References

(1) Park, Y.; Ahn, C.; Ahn, J.-G.; Kim, J. H.; Jung, J.; Oh, J.; Ryu, S.; Kim, S.; Kim, S. C.; Kim, T. Critical role of surface termination of sapphire substrates in crystallographic epitaxial growth of MoS2 using inorganic molecular precursors. *ACS nano* **2023**, *17* (2), 1196–1205.

(2) Li, T.; Guo, W.; Ma, L.; Li, W.; Yu, Z.; Han, Z.; Gao, S.; Liu, L.; Fan, D.; Wang, Z. Epitaxial growth of wafer-scale molybdenum disulfide semiconductor single crystals on sapphire. *Nat. Nanotechnol.* **2021**, *16* (11), 1201–1207.

(3) Fu, J.-H.; Min, J.; Chang, C.-K.; Tseng, C.-C.; Wang, Q.; Sugisaki, H.; Li, C.; Chang, Y.-M.; Alnami, I.; Syong, W.-R. Oriented lateral growth of two-dimensional materials on c-plane sapphire. *Nat. Nanotechnol.* **2023**, *18* (11), 1289–1294.

(4) Shi, Y.; Yang, P.; Jiang, S.; Zhang, Z.; Huan, Y.; Xie, C.; Hong, M.; Shi, J.; Zhang, Y. Na-assisted fast growth of large single-crystal MoS_2 on sapphire. *Nanotechnology* **2018**, *30* (3), 034002.

(5) Xue, G.; Qin, B.; Ma, C.; Yin, P.; Liu, C.; Liu, K. Large-area epitaxial growth of transition metal dichalcogenides. *Chem. Rev.* **2024**, *124* (17), 9785–9865.

(6) Nascimento, V. B.; Moore, R.; Rundgren, J.; Zhang, J.; Cai, L.; Jin, R.; Mandrus, D.; Plummer, E. W. Procedure for LEED I-V structural analysis of metal oxide surfaces: Ca 1.5 Sr 0.5 RuO 4 (001). *Phys. Rev. B* **2007**, *75* (3), 035408.

(7) Jona, F.; Strozier Jr, J.; Yang, W. Low-energy electron diffraction for surface structure analysis. *Rep. Prog. Phys.* **1982**, *45* (5), 527.

(8) MacRae, A. Low-Energy Electron Diffraction: Improved experimental methods provide new information on the structure of surfaces of solids. *Science* **1963**, *139* (3553), 379–388.

(9) Walter, S.; Blum, V.; Hammer, L.; Müller, S.; Heinz, K.; Giesen, M. The role of an energy-dependent inner potential in quantitative low-energy electron diffraction. *Surf. Sci.* **2000**, *458* (1-3), 155–161.

(10) Saldin, D.; Spence, J. On the mean inner potential in high-and low-energy electron diffraction. *Ultramicroscopy* **1994**, *55* (4), 397–406.

(11) Dai, Z.; Jin, W.; Grady, M.; Sadowski, J. T.; Dadap, J. I.; Osgood Jr, R. M.; Pohl, K. Surface structure of bulk 2H-MoS_2 (0001) and exfoliated suspended monolayer MoS_2: A selected area low energy electron diffraction study. *Surf. Sci.* **2017**, *660*, 16–21.

(12) Van Hove, M.; Tong, S.; Elconin, M. Surface structure refinements of 2H-MoS_2, 2H-$NbSe_2$ and W(100) p(2× 1)-O via new reliability factors for surface crystallography. *Surf. Sci.* **1977**, *64* (1), 85–95.

(13) Kim, T. K. T test as a parametric statistic. *Korean journal of anesthesiology* **2015**, *68* (6), 540–546.

(14) Van Der Zande, A. M.; Huang, P. Y.; Chenet, D. A.; Berkelbach, T. C.; You, Y.; Lee, G.-H.; Heinz, T. F.; Reichman, D. R.; Muller, D. A.; Hone, J. C. Grains and grain boundaries in highly crystalline monolayer molybdenum disulphide. *Nat. Mater.* **2013**, *12* (6), 554–561.

(15) Lachnitt, J. AQuaLEED software. *Private Communication*.

(16) Barbieri, A.; Van Hove, M. SATLEED package. *private communication*.

(17) Jugovac, M.; Menteş, T. O.; Genuzio, F.; Lachnitt, J.; Feyer, V.; Flege, J. I.; Locatelli, A. Sensitivity to crystal stacking in low-energy electron microscopy. *Appl. Surf. Sci.* **2021**, *566*, 150656.

(18) Flege, J. I.; Lachnitt, J.; Mazur, D.; Sutter, P.; Falta, J. Role of RuO_2 (100) in surface oxidation and CO oxidation catalysis on Ru (0001). *Phys. Chem. Chem. Phys.* **2016**, *18* (1), 213–219.

(19) Ganta, D.; Sinha, S.; Haasch, R. T. 2-D material molybdenum disulfide analyzed by XPS. *Surf. Sci. Spectra* **2014**, *21* (1), 19–27.
